# Effects of Dexmedetomidine Versus Ketorolac as Local Anesthetic Adjuvants on the Onset and Duration of Infraclavicular Brachial Plexus Block

**DOI:** 10.5812/aapm.17620

**Published:** 2014-08-02

**Authors:** Alireza Mirkheshti, Asadollah Saadatniaki, Alireza Salimi, Alireza Manafi Rasi, Elham Memary, Habiballah Yahyaei

**Affiliations:** 1 Anesthesiology Department, Shahid Beheshti University of Medical Sciences, Tehran, Iran; 2 Orthopedic Department, Shahid Beheshti University of Medical Sciences, Tehran, Iran

**Keywords:** Ketorolac, Dexmedtomidine, Infraclavicular Brachial Plexus

## Abstract

**Background:**

Infraclavicular brachial plexus block is an appropriate approach for distal arm and forearm surgeries. Local anesthetic adjuvant agents are used to improve the quality of nerve blocks. Dexmedetomidine and ketorolac are two different types of adjuvants, which have been used in some studies.

**Objectives:**

The purpose of this study was to examine the effects of dexmedetomidine and ketorolac as local anesthetic adjuvants on the onset and duration of infraclavicular brachial plexus block under ultrasound guide technique.

**Patients and Methods:**

In a clinical trial study, 111 ASA class I and II patients who were candidates for elective distal arm and forearm surgeries under ultrasound guided infraclavicular brachial plexus block divided into three 37 patient groups. In dexmedetomidine group, 25 mL of lidocaine 1.5% plus 4 ml of normal saline and 100 mcg of dexmedetomidine was injected. Ketorolac group received 25 mL of Lidocaine 1.5% plus 5 mL of ketorolac, and placebo group received 25 mL of lidocaine 1.5% plus 5 mL of normal saline as local anesthetic solution. Sensory and motor onset blocks, duration of sensory and motor blocks and first time to analgesic request and hemodynamic parameters were all recorded.

**Results:**

There were no significant differences in sensory block onset between three groups (P = 0.177). Motor block onset was statistically less in dexmedetomidine compared to ketorolac and placebo groups (both Ps < 0.001). Sensory block duration in dexmedetomidine group was significantly longer than ketorolac and placebo groups (both Ps < 0.001). Motor block duration in dexmedetomidine group was significantly longer than ketorolac and placebo groups (both Ps < 0.001). Time to first analgesic request after the procedures was longer in ketorolac compared to dexmedetomidine and placebo groups (P = 0.016, P < 0.001 respectively), but it was longer in dexmedetomidine compared to placebo group (P = 0.003). The differences of diastolic blood pressure in-between the 5th to 140th minutes after local anesthetic injection among the 3 groups were statistically significant and dexmedetomidine group shows the most reduction in diastolic blood pressure (P < 0.001). Dexmedetomidine showed the lowest mean arterial pressure (P = 0.016) and heart rate in dexmedetomidine group was significantly lower than ketorolac and placebo groups (P = 0.043).

**Conclusions:**

Our study showed that dexmedetomidine had better effects on sensory and motor block duration and motor block onset in comparison with ketorolac, as lidocaine adjuvants in infraclavicular brachial plexus block were present in both protocols. However, the first time to analgesic request by ketorolac was longer than dexmedetomidine.

## 1. Background

Infraclavicular brachial plexus block is a popular approach in accomplishing upper extremity anesthesia, which is well suited for distal arm and forearm surgeries. There are several techniques for infraclavicular nerve block by using nerve stimulation or ultrasound. Adjuvant agents are wide ranges of drugs, which is co-administered with local anesthetics to improve the speed of onset and quality or duration of nerve blocks. Dexmedetomidine is an agonist of ɑ2 adrenergic receptors that in some trials, reduced the onset time and improved the duration of sensory and motor blocks (1-3); but in the other ones, it had no effect or even showing a delay in the onset and duration of sensory and motor blocks (4, 5). Eventhough,dexmedetomidine is a potential local anesthetic adjuvant with facilitative effects as a part of peripheral nerve blocks, the data regarding its complete safety on human beingsare insufficient (6). Ketorolac is a parenteral non-steroidal anti-inflammatory drug, which provides analgesia by inhibiting prostaglandin synthesis. It has been shown that ketorolac - as an adjuvant to local anesthetics - produces longer duration and better quality of analgesia during peripheral nerve block (7).

## 2. Objectives

In this study we compared dexmedetomidine and ketorolac effects as local anesthetic adjuvants upon the onset and duration of sensory and motor nerve blocks under ultrasound guided infraclavicular brachial plexus block. Furthermore, we assessed hemodynamic parameters and time to first analgesic request of patients by distal arm and forearm surgeries.

## 3. Patients and Methods

After obtaining written informed consent from each patient and approval of institutional review board, 111 ASA classes I and II patients who were candidates for elective distal arm and forearm surgeries for less than 2.5 hours were selected and entered into our double-blinded randomized clinical trial. The exclusion criteria of our study comprised: any allergic reactions to NSAIDS, lidocaine and α2 agonists, all patients with hypertension, cardiac, hepatic or renal diseases, the patients who were under treatment of any α2 agonist or antagonist agents, pregnant women, drug abusers and psychiatric patients. Moreover, every patient who had anatomical or vascular abnormality in the upper extremity was excluded from our survey. By considering the statistical power of 95% and type one error of α = 5%, 111 patients were included and then divided into 3 groups by permuted block randomization, which every group consists of 37 patients as: Dexmedetomidine (D), Ketorolac (K) and Control (C) groups. All patients were monitored with electrocardiogram, pulse oximetry and noninvasive blood pressure monitoring at the time of their entrance to the operating room and throughout the procedures. They received midazolam 0.02 mg/kg and fentanyl 1 μg/kg intravenously as premedication.Infraclavicular brachial plexus blocks were performed by using ultrasound (SonoAce Pico, Samsung Medison Ultrasound machine, South Korea) and nerve stimulation (B Braun nerve stimulator, USA) techniques. Patients were in supine position by the head turning slightly to the opposite side of their blocks. The injured hand was placed on the patient’s abdomen and the shoulder was moved downward as much as possible .The parasagittal linear probe (8-14 MHz) by sterile sleeve was positioned near the coracoid process to identify the axillary artery. Then, the attempt was made to identify lateral, medial and posterior cords of brachial plexus, based on their positions relativeto the artery. After injecting of lidocaine 2% by a 2 mL syringe for anesthetizing the skin, a 50 mm and gauge 22 stimulating needle (B Braun, Germany) attached to nerve stimulator (0.8 to 0.5 mA, 0.1 ms) was used to anesthetized each cord. In dexmedetomidine group, 30 mL of a solution containing 25 mL of lidocaine1.5% plus 4 mL of normal saline and 100 µg of dexmedetomidine (Precedex 200 µg/2 mL, Hospira, USA) was injected by the amount of 10 mL for each cord. In ketorolac group, 30 mL of a solution containing 25 mL of lidocaine 1.5%, plus 5 mL of ketorolac (ketorolac trometamol, 10 mg/1 mL, Roche, UK) was injected as the mentioned amount. In control group, 30 mL of a solution containing 25 mL of lidocaine 1.5% plus 5 mL of normal saline was injected to anesthetize all three cords as 10 mL for every cord.

Sensory block was assessed by the loss to pinprick through a 22 gauge needle every 30 seconds. If the patient could not recognize the pinprick in all dermatomes related to musculocutaneous, median, ulnar and radial nerves, the time was recorded as sensory onset block. The duration between this time and the time of sense of pinprick in all related upper extremity dermatomes was recorded as duration of sensory block. All flexion and extension of elbow, wrist and fingers related to the function of musculocutaneous, median, ulnar and radial nerves were evaluated every 30 seconds after local anesthetic injection and the motor onset block was recorded when all mentioned movements were disappeared. The duration between this time and returning all flexion and extension movements was assessed as motor block duration. Systolic, diastolic and mean blood pressures, heart rate and saturation of Oxygen by pulse-oximetry were all recorded in the time of injection, 5th, 10th, 15th and 20th minutes and then every 10 minute till the end of operation. Meanwhile, time to first analgesic request according to visual analogue scale (VAS) more than 3 was recorded. Post operation pain was managed with repeated doses of morphine (2 mg) to VAS 3 or less. Statistical analysis was performed by SPSS (version 21.0, IBM Co. Chicago, IL). Normal distribution of data was investigated by Kolmogorov-Smirnov test and Q-Q plot. To compare the results of the groups, we used Kruskall-Wallis test. In the last step, to compare the results throughout the study, we used GeneralizedLinear Mixed model (GLMM).Two by two comparisons of groups was adjusted for multiple comparisons by Bonferroni method. P values less than 0.05 were considered as statistically significant.

## 4. Results

**Figure 1.**
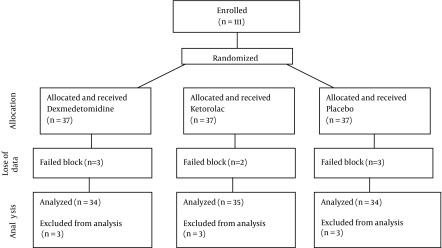
Consort Diagram Showing the Flow of Participants

As the consort diagram shows, 111 patients enrolled in the study, however 8 cases were excluded fromour survey due to incomplete nerve block and we had to consider general anesthesia as the second plan. Thus, data analysis was performed on 103 patients (Figure 1).

Characteristics of patients are shown in Table 1 and the sensory and motor block onsets and duration of sensory and motor blocks data all are depicted in Table 2. No significant differences was seen in sensory block onset between three groups (P = 0.177). Motor block onset was statistically shorter in dexmedetomidine, compared to ketorolac and placebo groups (both P < 0.001). Sensory block duration in dexmedetomidine group was significantly longer than ketorolac and placebo groups (all Ps < 0.001). Motor block duration in dexmedetomidine group was significantly longer than ketorolac and placebo groups (both Ps < 0.001). Time to first analgesic request after the procedures according to VAS > 3 was longer in ketorolac compared to dexmedetomidine and placebo groups (P = 0.016, P < 0.001 respectively), but it was longer in dexmedetomidine compared to placebo group (P = 0.003) (Table 2).

**Table 1.** Characteristics of Patients in Three Groups ^a^

Parameter	Total	Dexmedetomidine	Ketorolac	Lidocaine	P Value
**Age, y**					0.990 ^b^
Mean ± SD	39 ± 14	39 ± 14	39 ± 15	39 ± 15	
Median (range)	42 (16-59)	41 (18-59)	39 (17-58)	43 (16-58)	
**Weight, kg**					0.363 ^b^
Mean ± SD	72 ± 10	72 ± 9	73 ± 10	70 ± 10	
Median (range)	71 (50-100)	72 (50-90)	75 (53-100)	70 (50-97)	
**Sex, No. (%)**					0.104 ^c^
Male	63 (61.2)	25 (73.5)	17 (48.6)	21 (61.8)	
Female	40 (38.8)	9 (26.5)	18 (51.4)	13 (38.2)	
**Systolic BP**					0.472 ^b^
Mean ± SD	130 ± 18	129 ± 18	132 ± 19	128 ± 18	
Median (range)	127 (97-189)	126 (103-189)	131 (97-166)	122 (102-169)	
**Diastolic BP**					0.035 ^b^
Mean ± SD	78.05 ± 12.5	73.94 ± 10.82	79.91 ± 12.58	80.29 ± 13.28	
Median (range)	77 (51-110)	72 (53-110)	80 (51-110)	77 (52-109)	
**MAP**					0.131 ^b^
Mean ± SD	95.29 ± 13.08	92.2 ± 11.8	97.33 ± 13.26	96.35 ± 13.89	
Median (range)	93 (70 to 126)	88.5 (72.33-124.67)	96.33 (73-123.33)	93 (70-126)	
**HR**					0.255 ^b^
Mean ± SD	83 ± 12	83 ± 9	81 ± 14	85 ± 13	
Median (range)	84 (57-130)	86 (60-97)	80 (57-130)	86 (66-109)	
**SPO**_**2**_					0.091 ^b^
Mean ± SD	96 ± 2	97 ± 2	96 ± 2	97 ± 2	
Median (range)	96 (92-100)	97 (92-99)	96 (92-98)	96 (94-100)	

^a^ Abbreviations: BP, blood pressure; MAP, mean arterial pressure; HR, heart rate; SPO_2_, s pulse oximeter saturation of O_2_.

^b^ Based on Kruskall-Wallis test.

^c^ Based on Chi-Square test.

**Table 2.** Comparison of Infraclavicular Block Characteristics

Parameter	Dexmedtomidine	Ketorolac	Lidocaine	P Value ^a^
**Sensory block onset**				0.177
Mean ± SD	8.68 ± 3.57	11.79 ± 3.09	11.91 ± 4.66	
Median (range)	8 (4-17)	11 (7-20)	10.5 (5-25)	
**Motor block onset**				< 0.001
Mean ± SD	11.68 ± 4.3	15.94 ± 3.67	17.35 ± 5.46	
Median (range)	10 (7-25)	16 (9-27)	17.5 (6-30)	
**Sensory block duration**				
Mean ± SD	180 ± 74	132 ± 54	137 ± 92	< 0.001
Median (range)	151 (41 to 372)	114 (47-297)	115 (65-480)	
**Motor block duration**				< 0.001
Mean ± SD	170 ± 67	109 ± 50	117 ± 90	
Median (range)	140 (80-360)	98 (40-292)	106 (70-450)	
**first Time to analgesic request**				< 0.001
Mean ± SD	217 ± 81	315 ± 145	180 ± 95	
Median (range)	188 (47-402)	291 (107-620)	162 (91-510)	

^a^ Based on Kruskall-Wallis test.

**Figure 2.**
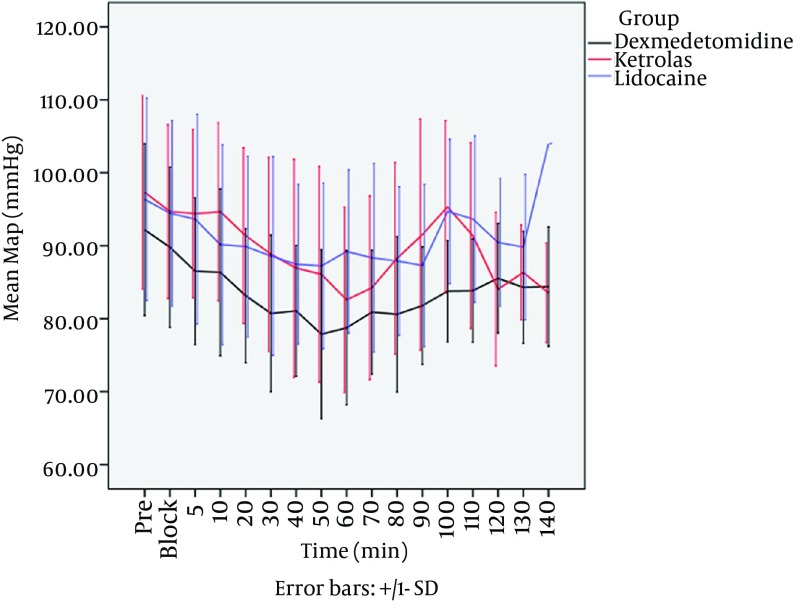
Mean Arterial Blood Pressure in Three Group

**Figure 3.**
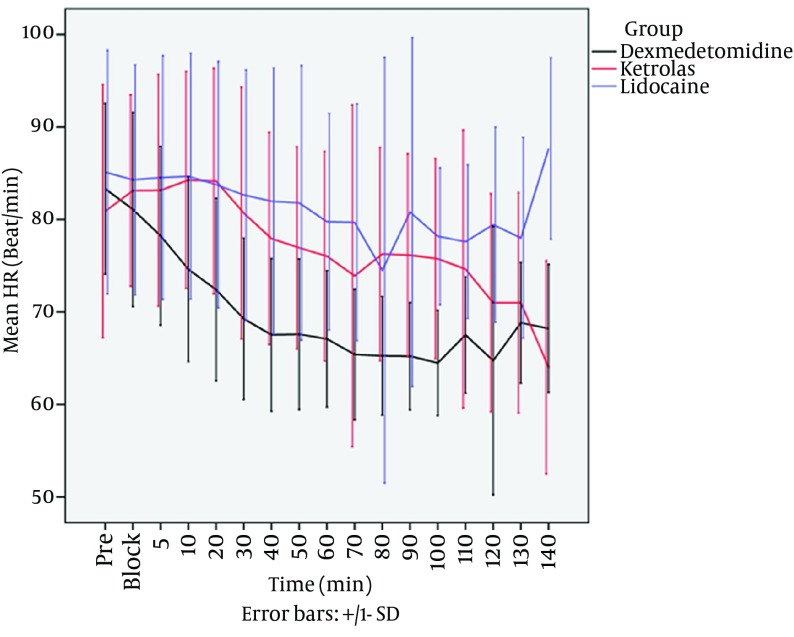
Mean Heart rate Between Three Groups

The difference of mean systolic blood pressure was not statistically significantamong the 3 groups during the procedures (P = 0.476). The results of mixed regression model show that after considering the basic effects of mean diastolic blood pressure, the differences between diastolic pressure in 3 groups between 5th to 140th minutes after local anesthetic injection were statistically significant, Dexmedetomidine group shows the most reduction in diastolic blood pressure (P < 0.001). The results of mixed regression model showed that every group had a decrease in mean arterial pressure (MAP), but dexmedetomidine group showed the lowest MAP during the procedures (P = 0.016) (Figure 2). Heart rate in dexmedetomidine group was significantly lower than ketorolac and placebo groups, even by considering the basic effect of heart rates between 3 groups (P = 0.043) (Figure 3). There were no side effects in each group.

## 5. Discussion

The purpose of the study was to assess the efficacy of dexmedetomidine and ketorolac as two different types of local anesthetic adjuvants in infraclavicular brachial plexus block. Our study demonstrated that the duration of motor and sensory blocks by dexmedetomidine were longer than Ketorolac. Our results about the increased duration of sensory and motor blocks by Dexmedetomidine as local anesthetic adjuvants are in agreement with the previous studies in different peripheral and neuraxial nerve blocks (8-14). Our results about ketorolac effects as local anesthetic adjuvant on duration of sensory and motor block support Budnyuk et al. study, in which ketorolac could not increase the duration of sensory and motor blocks when added to bupivacaine in brachial plexus block (15). The present study showed that ketorolac could not decrease sensory and motor block onset in infraclavicular brachial plexus block. The results about ketorolac on sensory and motor block onset provides more evidence for Budnyuk et al. study (15). However, our findings about sensory block onsets by dexmedetomidine are in disagreement with some studies (3, 11, 12, 14), which showed it could not decrease the onset time. One possible explanation for this disagreement on sensory block onset is that they performed their surveys by using bupivacaine as the main local anesthetic; but we used lidocaine as our main local anesthetic, which may cause different effects on sensory block onset. Our findings about motor block onset showed that dexmedetomidine decreased motor block onset compared to ketorolac and placebo, which are in agreement with Esmaoglu et al. (2) and Ammar et al. (11) showing that dexmedetomidine decrease motor onset time but the results are in disagreement with Kaygusuz study (9), in which dexmedetomidine did not decrease motor onset block. Our study showed that the time to first analgesic request by both ketorolac and dexmedetomidine increased. But, this increased time by ketorolac was more significant than dexmedetomidine. The results about the first time to analgesic request by both drugs support the previous studies (13, 14, 16). The present study showed dexmedetomidine decreased mean arterial and diastolic blood pressures and heart rate during the procedures, which are in agreement with the other studies (4, 5, 9, 11). Our data about ketorolac effects also support the previous data (17).

The main limitation of this study was that it was not possible for us to evaluate neurologic complications caused by dexmedetomidine or ketorolac. In some studies dexmedetomidine produced deleterious effects on neural system (18), but in the others, the adverse effects have not seen yet (14, 19). We recommend further studies focusing in adverse effects of perineural injection of these drugs. We saw no immediate side effect in this study, but intravascular injection of local anesthetics and pneumothorax were reported in other studies (20, 21).

Our study showed that dexmedetomidine had better effects on sensory and motor block duration and motor block onset in comparison with ketorolac, as lidocaine adjuvants in infraclavicular brachial plexus block were present in both protocols. However, the first time to analgesic request by ketorolac was longer than dexmedetomidine. 
